# Supplementary material for: The effect of adding neuromuscular electrical stimulation to exercise therapy on patellofemoral pain: A systematic review and meta-analysis
Source: PLoS One. 2025 Jun 23;20(6):e0326785. doi: 10.1371/journal.pone.0326785 (PMC12184933; doi:10.1371/journal.pone.0326785)
Supplement: S1 File — (DOCX) [file pone.0326785.s001.docx]

Search Strategies

Database: PubMed

Date searched: 01 July 2024

#1 Patellofemoral Pain Syndrome [Mesh Major Topic]

#2 Pain Syndrome, Patellofemoral OR Anterior Knee Pain Syndrome OR Patellofemoral Syndrome OR Patellofemoral Pain OR Pain, Patellofemoral OR Patellofemoral Pains

#3 #1 OR #2

#4 Electric Stimulation [Mesh Major Topic]

#5 Electric Stimulation Therapy [Mesh Major Topic]

#6 (Electrical AND Stimulation) OR (Electrical AND Stimulations) OR (Stimulation, Electrical) OR (Stimulations, Electrical) OR (Stimulation, Electric) OR (Electric AND Stimulations) OR (Stimulations, Electric) OR (Therapeutic AND Electrical AND Stimulation) OR (Electrical AND Stimulation, Therapeutic) OR (Stimulation, Therapeutic AND Electrical) OR (Therapeutic AND Electric AND Stimulation) OR (Electric AND Stimulation, Therapeutic) OR (Stimulation, Therapeutic AND Electric) OR (Electrical AND Stimulation AND Therapy) OR (Stimulation Therapy, Electrical) OR (Therapy, Electrical AND Stimulation) OR (Therapy, Electric AND Stimulation) OR (Stimulation AND Therapy, Electric) OR Electrotherapy OR (Interferential AND Current AND Electrotherapy) OR (Electrotherapy, Interferential AND Current) OR EMS OR NMES

#7 #4 OR #5 OR #6

#8 #3 AND #7

#9 ((((((randomized controlled trial [pt]) OR (controlled clinical trial [pt])) OR (randomized [tiab])) OR (placebo [tiab])) OR (clinical trials as topic [mesh: noexp])) OR (randomly [tiab])) OR (trial [ti])

#10 animals [mh] NOT humans [mh]

#11 #9 NOT #10

#12 #8 AND #11

Database: Embase

Date searched: 01 July 2024

#1 'patellofemoral pain'/exp

#2 'patellofemoral pain syndrome'/exp

#3 'patella femoral pain': ab,ti OR 'patellar femoral pain': ab,ti OR 'patello-femoral pain': ab,ti OR 'patellofemoral pain': ab,ti OR 'patella femoral pain syndrome': ab,ti OR 'patellar femoral pain syndrome': ab,ti OR 'patello-femoral pain syndrome': ab,ti OR 'patello-femoral stress syndrome': ab,ti OR 'patello-femoral syndrome': ab,ti OR 'patellofemoral stress syndrome': ab,ti OR 'patellofemoral syndrome': ab,ti OR 'patellofemoral pain syndrome': ab,ti

#4 #1 OR #2 OR #3

#5 'electrostimulation'/exp

#6 'electrotherapy'/exp

#7 'neuromuscular electrical stimulation'/exp

#8 'Electrical AND Stimulation':ab,ti OR 'Electrical AND Stimulations':ab,ti OR 'Stimulation, Electrical':ab,ti OR 'Stimulations, Electrical':ab,ti OR 'Stimulation, Electric':ab,ti OR 'Electric AND Stimulations':ab,ti OR 'Stimulations, Electric':ab,ti OR 'Therapeutic AND Electrical AND Stimulation':ab,ti OR 'Electrical AND Stimulation, Therapeutic':ab,ti OR 'Stimulation, Therapeutic AND Electrical':ab,ti OR 'Therapeutic AND Electric AND Stimulation':ab,ti OR 'Electric AND Stimulation, Therapeutic':ab,ti OR 'Stimulation, Therapeutic AND Electric':ab,ti OR 'Electrical AND Stimulation AND Therapy':ab,ti OR 'Stimulation Therapy, Electrical':ab,ti OR 'Therapy, Electrical AND Stimulation':ab,ti OR 'Therapy, Electric AND Stimulation':ab,ti OR 'Stimulation AND Therapy, Electric':ab,ti OR 'Electrotherapy':ab,ti OR 'Interferential AND Current AND Electrotherapy':ab,ti OR 'Electrotherapy, Interferential AND Current':ab,ti OR 'EMS':ab,ti OR 'NMES':ab,ti

#9 #5 OR #6 OR #7 OR #8

#10 'randomized controlled trial'/exp

#11 #4 AND #9 AND #10

Database: Web of Science

Date searched: 01 July 2024

#1

T1= (Patellofemoral pain) OR AB= (Patellofemoral pain) OR TI= (Pain Syndrome, Patellofemoral OR Anterior Knee Pain Syndrome OR Patellofemoral Syndrome OR Patellofemoral Pain OR Pain, Patellofemoral OR Patellofemoral Pains) OR AB= (Pain Syndrome, Patellofemoral OR Anterior Knee Pain Syndrome OR Patellofemoral Syndrome OR Patellofemoral Pain OR Pain, Patellofemoral OR Patellofemoral Pains)

#2

#2

TI=((Electrical AND Stimulation) OR (Electrical AND Stimulations) OR (Stimulation, Electrical) OR (Stimulations, Electrical) OR (Stimulation, Electric) OR (Electric AND Stimulations) OR (Stimulations, Electric) OR (Therapeutic AND Electrical AND Stimulation) OR (Electrical AND Stimulation, Therapeutic) OR (Stimulation, Therapeutic AND Electrical) OR (Therapeutic AND Electric AND Stimulation) OR (Electric AND Stimulation, Therapeutic) OR (Stimulation, Therapeutic AND Electric) OR (Electrical AND Stimulation AND Therapy) OR (Stimulation Therapy, Electrical) OR (Therapy, Electrical AND Stimulation) OR (Therapy, Electric AND Stimulation) OR (Stimulation AND Therapy, Electric) OR Electrotherapy OR (Interferential AND Current AND Electrotherapy) OR (Electrotherapy, Interferential AND Current) OR EMS OR NMES OR (Neuromuscular AND Electrical AND Stimulation)) OR AB=((Electrical AND Stimulation) OR (Electrical AND Stimulations) OR (Stimulation, Electrical) OR (Stimulations, Electrical) OR (Stimulation, Electric) OR (Electric AND Stimulations) OR (Stimulations, Electric) OR (Therapeutic AND Electrical AND Stimulation) OR (Electrical AND Stimulation, Therapeutic) OR (Stimulation, Therapeutic AND Electrical) OR (Therapeutic AND Electric AND Stimulation) OR (Electric AND Stimulation, Therapeutic) OR (Stimulation, Therapeutic AND Electric) OR (Electrical AND Stimulation AND Therapy) OR (Stimulation Therapy, Electrical) OR (Therapy, Electrical AND Stimulation) OR (Therapy, Electric AND Stimulation) OR (Stimulation AND Therapy, Electric) OR Electrotherapy OR (Interferential AND Current AND Electrotherapy) OR (Electrotherapy, Interferential AND Current) OR EMS OR NMES OR (Neuromuscular AND Electrical AND Stimulation))

#3 #1 AND #2

Database: the Cochrane Library

Date searched: 01 July 2024

#1 MeSH descriptor: [Patellofemoral Pain Syndrome] explode all trees

#2 MeSH descriptor: [Electric Stimulation] explode all trees

#3 MeSH descriptor: [Electric Stimulation Therapy] explode all trees

#4 (Synonyms) OR (Anterior Knee Pain Syndrome) OR (Pain Syndrome, Patellofemoral) OR (Patellofemoral Pain) OR (Patellofemoral Pains) OR (Pain, Patellofemoral) OR (Patellofemoral Syndrome)

#5 (Electrical AND Stimulation) OR (Electrical AND Stimulations) OR (Stimulation, Electrical) OR (Stimulations, Electrical) OR (Stimulation, Electric) OR (Electric AND Stimulations) OR (Stimulations, Electric) OR (Therapeutic AND Electrical AND Stimulation) OR (Electrical AND Stimulation, Therapeutic) OR (Stimulation, Therapeutic AND Electrical) OR (Therapeutic AND Electric AND Stimulation) OR (Electric AND Stimulation, Therapeutic) OR (Stimulation, Therapeutic AND Electric) OR (Electrical AND Stimulation AND Therapy) OR (Stimulation Therapy, Electrical) OR (Therapy, Electrical AND Stimulation) OR (Therapy, Electric AND Stimulation) OR (Stimulation AND Therapy, Electric) OR Electrotherapy OR (Interferential AND Current AND Electrotherapy) OR (Electrotherapy, Interferential AND Current) OR EMS OR NMES

#6 #1 OR #4

#7 #2 OR #3 OR #5

#8 #6 AND #7

Database: Scopus

Date searched: 01 July 2024

TITLE-ABS-KEY（(Patellofemoral Pain Syndrome) OR （patella femoral pain） OR (patellar femoral pain) OR (patello-femoral pain) OR (patellofemoral pain) OR (patella femoral pain syndrome) OR (patellar femoral pain syndrome) OR (patello-femoral pain syndrome) OR (patello-femoral stress syndrome) OR (patello-femoral syndrome) OR （patellofemoral stress syndrome） OR （patellofemoral syndrome）OR （patellofemoral pain syndrome））AND TITLE-ABS-KEY((Electrical AND Stimulation) OR (Electrical AND Stimulations) OR (Stimulation, Electrical) OR (Stimulations, Electrical) OR (Stimulation, Electric) OR (Electric AND Stimulations) OR (Stimulations, Electric) OR (Therapeutic AND Electrical AND Stimulation) OR (Electrical AND Stimulation, Therapeutic) OR (Stimulation, Therapeutic AND Electrical) OR (Therapeutic AND Electric AND Stimulation) OR (Electric AND Stimulation, Therapeutic) OR (Stimulation, Therapeutic AND Electric) OR (Electrical AND Stimulation AND Therapy) OR (Stimulation Therapy, Electrical) OR (Therapy, Electrical AND Stimulation) OR (Therapy, Electric AND Stimulation) OR (Stimulation AND Therapy, Electric) OR Electrotherapy OR (Interferential AND Current AND Electrotherapy) OR (Electrotherapy, Interferential AND Current) OR EMS OR NMES OR (Neuromuscular AND Electrical AND Stimulation)) AND TITLE-ABS-KEY((randomized controlled trial*) OR (controlled clinical trial*) OR (randomized) OR (placebo*) OR (clinical trial*) OR (random*) OR (trial*))
