# Supplementary material for: The effect of adding neuromuscular electrical stimulation to exercise therapy on patellofemoral pain: A systematic review and meta-analysis
Source: PLoS One. 2025 Jun 23;20(6):e0326785. doi: 10.1371/journal.pone.0326785 (PMC12184933; doi:10.1371/journal.pone.0326785)
Supplement: S2 File — (PDF) [file pone.0326785.s002.pdf]

Author(s):
Question: NMSE compared to Placebo for PFPS
Setting:
Bibliography: . neuromuscular electrical stimulation for patellofemoral pain syndrome.

Certainty assessment							№ of patients		Effect		Certainty	Importance
№ of studies	Study design	Risk of bias	Inconsistency	Indirectness	Imprecision	Other considerations	NMSE	Placebo	Relative (95% CI)	Absolute (95% CI)		
**VAS**												
9	randomised trials	serious[a]	not serious	not serious	serious[b]	none	167	166	-	MD **0.33 lower** (0.54 lower to 0.13 lower)	⊕⊕◯◯ Low	
**AKPS**												
8	randomised trials	serious[a]	not serious	not serious	serious[b]	none	153	147	-	MD **4.47 higher** (2.85 higher to 6.09 higher)	⊕⊕◯◯ Low	
**Quadriceps Muscle Strength**												
4	randomised trials	very serious[c]	not serious	not serious	serious[b]	none	59	58	-	SMD **0.75 higher** (0.37 higher to 1.12 higher)	⊕◯◯◯ Very low	
**VAS-<1 month**												
5	randomised trials	not serious	very serious[d]	not serious	serious[b]	none	87	89	-	MD **0.67 lower** (1.34 lower to 0.01 higher)	⊕◯◯◯ Very low	
**VAS-1-3month**												
6	randomised trials	very serious[c]	not serious	not serious	serious[b]	none	133	133	-	MD **0.28 lower** (0.54 lower to 0.02 lower)	⊕◯◯◯ Very low	
**AKPS - 1-3month**												
6	randomised trials	very serious[c]	serious[e]	not serious	serious[b]	none	127	124	-	MD **4.32 higher** (1.8 higher to 6.84 higher)	⊕◯◯◯ Very low	
**Quadriceps Muscle Strength - <1 month**												
2	randomised trials	not serious	not serious	not serious	very serious[f]	none	26	26	-	SMD **0.64 higher** (0.08 higher to 1.2 higher)	⊕⊕◯◯ Low	
**Quadriceps Muscle Strength - 1-3month**												
2	randomised trials	not serious	not serious	not serious	very serious[f]	none	33	32	-	SMD **0.83 higher** (0.32 higher to 1.34 higher)	⊕⊕◯◯ Low	
**VMO/VL Ratio**												
2	randomised trials	not serious	very serious[g]	not serious	very serious[f]	none	27	27	-	SMD **0.8 higher** (0.33 lower to 1.93 higher)	⊕◯◯◯ Very low	

CI: confidence interval; **MD:** mean difference; **SMD:** standardised mean difference

Explanations

a. Most of the information comes from moderate bias
b. Small sample size
c. Most of the information comes from high-risk bias
d. I^2=81% large heterogeneity
e. I^2=53% large heterogeneity
f. Very small sample size
g. I^2=74% large heterogeneity
