# Supplementary material for: The effect of adding neuromuscular electrical stimulation to exercise therapy on patellofemoral pain: A systematic review and meta-analysis
Source: PLoS One. 2025 Jun 23;20(6):e0326785. doi: 10.1371/journal.pone.0326785 (PMC12184933; doi:10.1371/journal.pone.0326785)
Supplement: S4 File — (DOCX) [file pone.0326785.s004.docx]

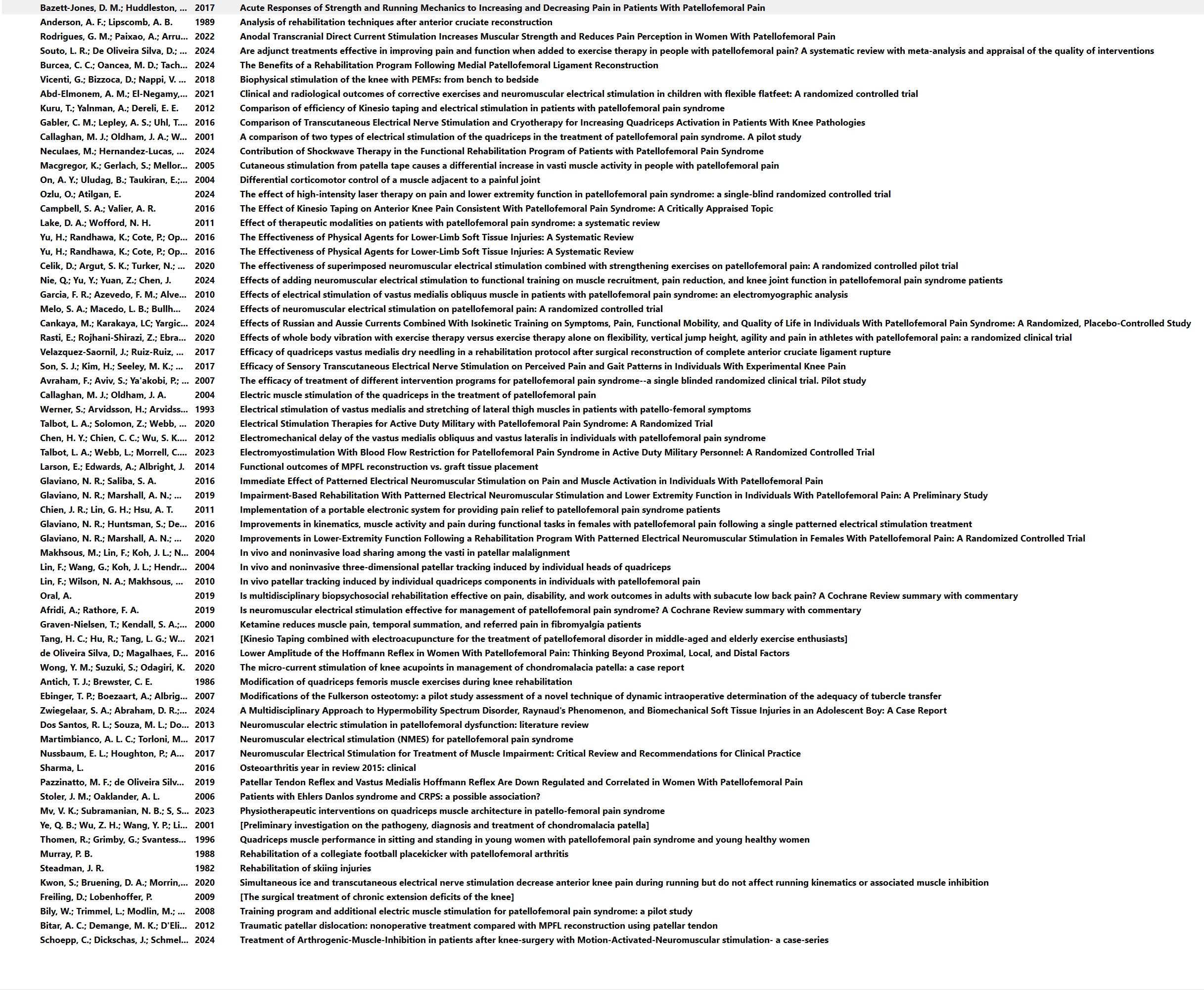
After keyword research and deletion of duplicates, the following 68 documents were collected:


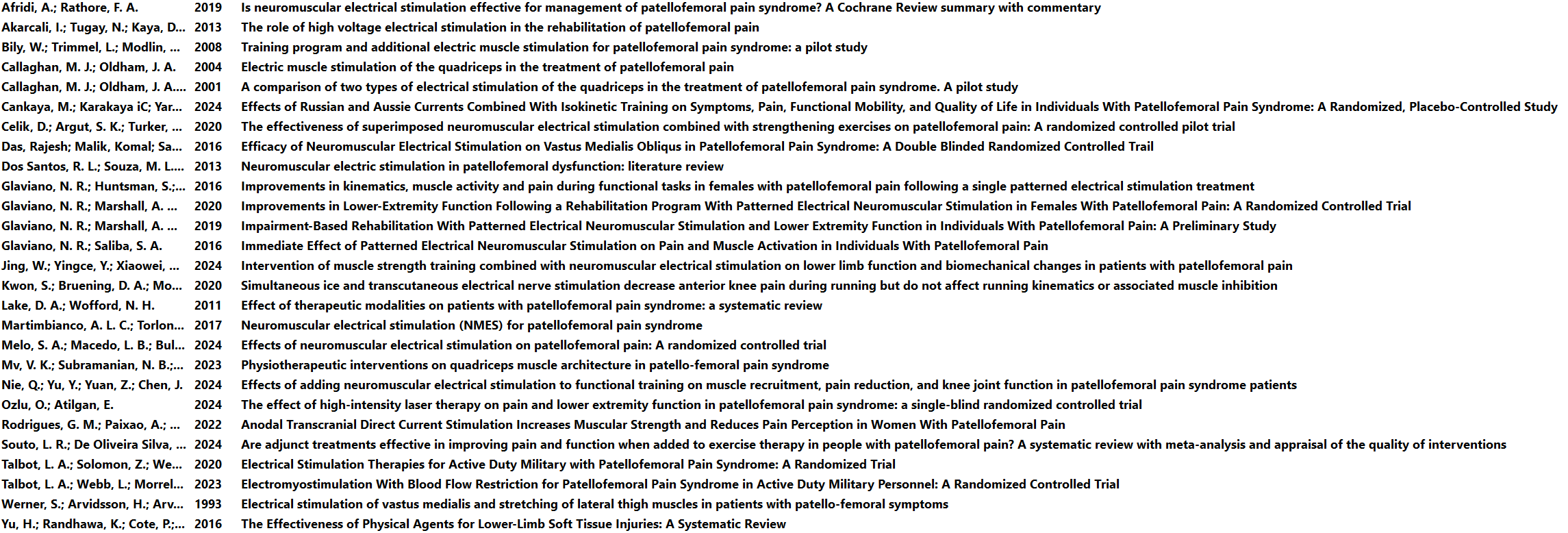
Based on the inclusion and exclusion criteria, after going through the titles and abstracts, a total of 27articles that provided full-text information were filtered as follows:

The following articles were further excluded based on the full text as well as inclusion and exclusion criteria:

The following 6 studies were excluded because they did not involve exercise therapy:

1. Rodrigues, G. M., Paixao, A., Arruda, T., de Oliveira, B. R. R., Maranhao Neto, G. A., Marques Neto, S. R., Lattari, E., & Machado, S. (2022). Anodal transcranial direct current stimulation increases muscular strength and reduces pain perception in women with patellofemoral pain. *Journal of Strength and Conditioning Research, 36*(2), 371-378.
2. Cankaya, M., iC, K., Yargi, P. M., & Karakaya, M. G. (2024). Effects of Russian and Aussie currents combined with isokinetic training on symptoms, pain, functional mobility, and quality of life in individuals with patellofemoral pain syndrome: A randomized, placebo-controlled study. *American Journal of Physical Medicine & Rehabilitation, 103*(11), 1017-1025.
3. Glaviano, N. R., & Saliba, S. A. (2016). Immediate effect of patterned electrical neuromuscular stimulation on pain and muscle activation in individuals with patellofemoral pain. *Journal of Athletic Training, 51*(2), 118-128.
4. Glaviano, N. R., Marshall, A. N., Mangum, L. C., Hart, J. M., Hertel, J., Russell, S., & Saliba, S. A. (2019). Impairment-based rehabilitation with patterned electrical neuromuscular stimulation and lower extremity function in individuals with patellofemoral pain: A preliminary study. *Journal of Athletic Training, 54*(3), 255-269.
5. Glaviano, N. R., Huntsman, S., Dembeck, A., Hart, J. M., & Saliba, S. (2016). Improvements in kinematics, muscle activity and pain during functional tasks in females with patellofemoral pain following a single patterned electrical stimulation treatment. *Clinical Biomechanics, 32*, 20-27.
6. Kwon, S., Bruening, D. A., Morrin, S. J., Kunz, D. M., Hopkins, J. T., & Seeley, M. K. (2020). Simultaneous ice and transcutaneous electrical nerve stimulation decrease anterior knee pain during running but do not affect running kinematics or associated muscle inhibition. *Clinical Biomechanics, 72*, 1-7.

The following 6 studies were excluded because they were reviews:

1. Souto, L. R., De Oliveira Silva, D., Pazzinatto, M. F., Siqueira, M. S., Moreira, R. F. C., & Serrao, F. V. (2024). Are adjunct treatments effective in improving pain and function when added to exercise therapy in people with patellofemoral pain? A systematic review with meta-analysis and appraisal of the quality of interventions. *British Journal of Sports Medicine, 58*(14), 792-804.
2. Lake, D. A., & Wofford, N. H. (2011). Effect of therapeutic modalities on patients with patellofemoral pain syndrome: a systematic review. *Sports Health, 3*(2), 182-189.
3. Yu, H., Randhawa, K., Cote, P., & Optima, C. (2016). The effectiveness of physical agents for lower-limb soft tissue injuries: A systematic review. *The Journal of Orthopaedic and Sports Physical Therapy, 46*(7), 523-554.
4. Afridi, A., & Rathore, F. A. (2019). Is neuromuscular electrical stimulation effective for management of patellofemoral pain syndrome? A Cochrane Review summary with commentary. *Musculoskeletal Science & Practice, 44*, 102059.
5. Dos Santos, R. L., Souza, M. L., & Dos Santos, F. A. (2013). Neuromuscular electric stimulation in patellofemoral dysfunction: literature review. *Acta Ortopédica Brasileira, 21*(1), 52-58.
6. Martimbianco, A. L. C., Torloni, M. R., Andriolo, B. N., Porfirio, G. J., & Riera, R. (2017). Neuromuscular electrical stimulation (NMES) for patellofemoral pain syndrome. *The Cochrane Database of Systematic Reviews, 12*(12), Cd011289.

The following 1 study was excluded because it was not a randomized controlled trial:

1. Werner, S., Arvidsson, H., Arvidsson, I., & Eriksson, E. (1993). Electrical stimulation of vastus medialis and stretching of lateral thigh muscles in patients with patello-femoral symptoms. *Knee Surgery, Sports Traumatology, Arthroscopy: Official Journal of the ESSKA, 1*(2), 85-92.

The following 4 studies were excluded because NMES was used in both the experimental and control groups:

1. Callaghan, M. J., & Oldham, J. A. (2004). Electric muscle stimulation of the quadriceps in the treatment of patellofemoral pain. *Archives of Physical Medicine and Rehabilitation, 85*(6), 956-962.
2. Callaghan, M. J., Oldham, J. A., & Winstanley, J. (2001). A comparison of two types of electrical stimulation of the quadriceps in the treatment of patellofemoral pain syndrome: A pilot study. *Clinical Rehabilitation, 15*(6), 637-646.
3. Ozlu, O., & Atilgan, E. (2024). The effect of high-intensity laser therapy on pain and lower extremity function in patellofemoral pain syndrome: A single-blind randomized controlled trial. *Lasers in Medical Science, 39*(1), 103.
4. Talbot, L. A., Webb, L., Morrell, C., Enochs, K., Hillner, J., Fagan, M., & Metter, E. J. (2023). Electromyostimulation with blood flow restriction for patellofemoral pain syndrome in active duty military personnel: A randomized controlled trial. *Military Medicine, 188*(7-8), e1859-e1868.

The following 1 study was excluded because the data in the article could not be extracted for analysis:

1. Melo, S. A., Macedo, L. B., Bullhoez, L. C. C., Cavalcanti, R. L., Azevedo Rodolfo, J. I., & Brasileiro, J. S. (2024). Effects of neuromuscular electrical stimulation on patellofemoral pain: A randomized controlled trial. *Journal of Bodywork and Movement Therapies, 39*, 390-397.

After screening, a total of 9 studies were included in the meta-analysis as follows:

1. Akarcali, I., Tugay, N., Kaya, D., Atay, A., & Doral, M. N. (2013). The role of high voltage electrical stimulation in the rehabilitation of patellofemoral pain. *The Pain Clinic, 14*(3), 207-212.
2. Bily, W., Trimmel, L., Modlin, M., Kaider, A., & Kern, H. (2008). Training program and additional electric muscle stimulation for patellofemoral pain syndrome: a pilot study. *Archives of Physical Medicine and Rehabilitation, 89*(7), 1230-1236.
3. Celik, D., Argut, S. K., Turker, N., & Kilicoglu, O. I. (2020). The effectiveness of superimposed neuromuscular electrical stimulation combined with strengthening exercises on patellofemoral pain: A randomized controlled pilot trial. *Journal of Back and Musculoskeletal Rehabilitation, 33*(4), 693-699.
4. Das, R., Malik, K., Sarkar, B., Saha, S., & Biswas, A. (2016). Efficacy of neuromuscular electrical stimulation on vastus medialis obliquus in patellofemoral pain syndrome: A double blinded randomized controlled trial. *International Journal of Therapies and Rehabilitation Research, 5*(5).
5. Glaviano, N. R., Marshall, A. N., Mangum, L. C., Hart, J. M., Hertel, J., Russell, S., & Saliba, S. (2020). Improvements in lower-extremity function following a rehabilitation program with patterned electrical neuromuscular stimulation in females with patellofemoral pain: A randomized controlled trial. *Journal of Sport Rehabilitation, 29*(8), 1075-1085.
6. Jing, W., Yingce, Y., Xiaowei, Y., Boshi, X., Jianbin, Z., Chen, Y., Tianfeng, L., & Zhipeng, Z. (2024). Intervention of muscle strength training combined with neuromuscular electrical stimulation on lower limb function and biomechanical changes in patients with patellofemoral pain. *Chinese Journal of Tissue Engineering Research, 28*(9), 1365-1371.
7. Mv, V. K., Subramanian, N. B., S, S., Kotamraju, S., & Krishnan, M. (2023). Physiotherapeutic interventions on quadriceps muscle architecture in patello-femoral pain syndrome. *Bioinformation, 19*(4), 454-459.
8. Nie, Q., Yu, Y., Yuan, Z., & Chen, J. (2024). Effects of adding neuromuscular electrical stimulation to functional training on muscle recruitment, pain reduction, and knee joint function in patellofemoral pain syndrome patients. *Medicine (Baltimore), 103*(3), e36095.
9. Talbot, L. A., Solomon, Z., Webb, L., Morrell, C., & Metter, E. J. (2020). Electrical stimulation therapies for active duty military with patellofemoral pain syndrome: A randomized trial. *Military Medicine, 185*(7-8), e963-e971.
