# Supplementary material for: The effect of adding neuromuscular electrical stimulation to exercise therapy on patellofemoral pain: A systematic review and meta-analysis
Source: PLoS One. 2025 Jun 23;20(6):e0326785. doi: 10.1371/journal.pone.0326785 (PMC12184933; doi:10.1371/journal.pone.0326785)
Supplement: S6 File — (DOCX) [file pone.0326785.s006.docx]

| 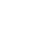 | **Random sequence generation (selection bias)** | **Allocation concealment (selection bias)** | **Blinding of participants and personnel (performance bias)** | **Blinding of outcome assessment (detection bias)** | **Incomplete outcome data (attrition bias)** | **Selective reporting (reporting bias)** | **Other bias** |
| --- | --- | --- | --- | --- | --- | --- | --- |
| Akarcali, N. Tugay 2002 | Low risk | Uncler risk | High risk | High risk | High risk | Low risk | Low risk |
| Col Laura A. Talbot 2020 | Low risk | Low risk | High risk | High risk | High risk | Low risk | Low risk |
| Derya Celik 2019 | Low risk | Low risk | High risk | Low risk | High risk | Low risk | Low risk |
| MV Vinaya Kumar 2023 | Low risk | Low risk | Low risk | Low risk | High risk | Low risk | Low risk |
| Neal R. Glaviano 2020 | Low risk | Low risk | Low risk | Low risk | Low risk | Low risk | Low risk |
| Qiu Nie, MM 2023 | Low risk | Low risk | High risk | Low risk | Low risk | Low risk | Low risk |
| Rajesh Kumar Das 2016 | Low risk | Low risk | Low risk | Low risk | High risk | Low risk | Low risk |
| Walter Bily 2008 | Low risk | Low risk | Uncler risk | High risk | High risk | Low risk | Low risk |
| Wu Jing 2024 | Low risk | Low risk | High risk | High risk | High risk | Low risk | Low risk |

**Author(s):**

**Question:** Experimental group compared to Control group for PFPS

**Setting:**

**Bibliography:** . neuromuscular electrical stimulation for patellofemoral pain syndrome.

| **Certainty assessment** | | | | | | | **№ of patients** | | **Effect** | | **Certainty** | **Importance** |
| --- | --- | --- | --- | --- | --- | --- | --- | --- | --- | --- | --- | --- |
| **№ of studies** | **Study design** | **Risk of bias** | **Inconsistency** | **Indirectness** | **Imprecision** | **Other considerations** | **Experimental group** | **Control group** | **Relative (95% CI)** | **Absolute (95% CI)** |  |  |
| **VAS** | | | | | | | | | | | | |
| 9 | randomised trials | serious^a^ | not serious | not serious | serious^b^ | none | 167 | 166 | - | MD **0.33 lower** (0.54 lower to 0.13 lower) | ⨁⨁◯◯ Low |  |
| **AKPS** | | | | | | | | | | | | |
| 8 | randomised trials | serious^a^ | not serious | not serious | serious^b^ | none | 153 | 147 | - | MD **4.47 higher** (2.85 higher to 6.09 higher) | ⨁⨁◯◯ Low |  |
| **Quadriceps Muscle Strength** | | | | | | | | | | | | |
| 4 | randomised trials | very serious^c^ | not serious | not serious | serious^b^ | none | 59 | 58 | - | SMD **0.75 higher** (0.37 higher to 1.12 higher) | ⨁◯◯◯ Very low |  |
| **VAS-<1 month** | | | | | | | | | | | | |
| 5 | randomised trials | not serious | very serious^d^ | not serious | serious^b^ | none | 87 | 89 | - | MD **0.67 lower** (1.34 lower to 0.01 higher) | ⨁◯◯◯ Very low |  |
| **VAS-1-3month** | | | | | | | | | | | | |
| 6 | randomised trials | very serious^c^ | not serious | not serious | serious^b^ | none | 133 | 133 | - | MD **0.28 lower** (0.54 lower to 0.02 lower) | ⨁◯◯◯ Very low |  |
| **AKPS - 1-3month** | | | | | | | | | | | | |
| 6 | randomised trials | very serious^c^ | serious^e^ | not serious | serious^b^ | none | 127 | 124 | - | MD **4.32 higher** (1.8 higher to 6.84 higher) | ⨁◯◯◯ Very low |  |
| **Quadriceps Muscle Strength - <1 month** | | | | | | | | | | | | |
| 2 | randomised trials | not serious | not serious | not serious | very serious^f^ | none | 26 | 26 | - | SMD **0.64 higher** (0.08 higher to 1.2 higher) | ⨁⨁◯◯ Low |  |
| **Quadriceps Muscle Strength - 1-3month** | | | | | | | | | | | | |
| 2 | randomised trials | not serious | not serious | not serious | very serious^f^ | none | 33 | 32 | - | SMD **0.83 higher** (0.32 higher to 1.34 higher) | ⨁⨁◯◯ Low |  |
| **VMO/VL Ratio** | | | | | | | | | | | | |
| 2 | randomised trials | not serious | very serious^g^ | not serious | very serious^f^ | none | 27 | 27 | - | SMD **0.8 higher** (0.33 lower to 1.93 higher) | ⨁◯◯◯ Very low |  |

**CI:** confidence interval; **MD:** mean difference; **SMD:** standardised mean difference

#### Explanations

a. Most of the information comes from moderate bias

b. Small sample size

c. Most of the information comes from high-risk bias

d. I²=81% large heterogeneity

e. I²=53% large heterogeneity

f. Very small sample size

g. I²=74% large heterogeneity
