# Supplementary material for: The effect of adding neuromuscular electrical stimulation to exercise therapy on patellofemoral pain: A systematic review and meta-analysis
Source: PLoS One. 2025 Jun 23;20(6):e0326785. doi: 10.1371/journal.pone.0326785 (PMC12184933; doi:10.1371/journal.pone.0326785)
Supplement: Attachment — Submitted filename: Response to Reviewers.docx [file pone.0326785.s009.docx]

**Response to reviewers**

**Dear reviewers:**

On behalf of my co-authors, we thank you very much for your letter and comments on our manuscript entitled " The effect of adding neuromuscular electrical stimulation to exercise therapy on patellofemoral pain: A systematic review and meta-analysis" (Manuscript ID: PONE-D-24-36303R1). We appreciate the editors and reviewers for their constructive and valuable comments. We have revised our manuscript considerably according to the editors’ and reviewers’ comments, questions, and suggestions. In the event that we missed any one of the comments please let us know. This document includes our responses to reviewers and editor comments point by point, and the revised portion are marked in red in our manuscript.

**Reply to reviewer #1**

**Comment 1:** I appreciate the authors for thoroughly addressing the comments. The manuscript has improved significantly; however, a few minor inconsistencies remain that would benefit from further revision. In particular, the Results section contains several structural and grammatical issues that should be carefully reviewed and corrected to enhance clarity and readability.

**Reply 1:** We sincerely thank the reviewer for their constructive feedback and encouraging assessment of our revised manuscript. We are pleased to hear that our previous revisions have significantly improved the paper. We acknowledge the reviewer's observation regarding minor inconsistencies in the Results section. We have carefully reviewed this section again and corrected all issues to enhance clarity and readability. We appreciate the reviewer's attention to detail, which has helped us further refine the quality of our manuscript

**Comment 2**: In lines 245-248, the authors should consider replacing the term "superimposed NMES" with "NMES combined with exercise" for clarity and consistency. The term "superimposed" may be ambiguous to some readers, whereas "combined with exercise" more clearly communicates the intervention strategy.

**Reply 2**: Thank you for your careful guidance. We agree that the term "superimposed NMES" may be ambiguous to some readers. As per your recommendation, we have replaced this term with "NMES combined with exercise" throughout the manuscript to improve clarity and maintain consistency in describing our intervention strategy.

**Page 13, line 231 in red**: NMES combined with exercise significantly reduced pain compared to controls

**Comment 3**: The sentence in lines 248-251 is structurally flawed and difficult to follow due to misplaced and redundant elements. Specifically, the phrase "suggest that (I²=81%) indicated..." is grammatically incorrect and confusing. The use of both “suggest” and “indicated” creates unnecessary duplication, and removing one of these words would improve the structure of the sentence and improve comprehensibility.

**Reply 3**: We appreciate the reviewer's careful assessment of our manuscript. We agree that the sentence in lines 248-251 contained redundant elements with both 'suggest that' and 'indicated' creating an awkward structure. We have revised the sentence to eliminate this duplication, improving clarity and readability.

**Page 13, line 232-235 in red**: In subgroup analyses, very low-certainty evidence and considerable statistical heterogeneity (I²=81%) suggested no significant effect when the intervention was less than or equal to 4 weeks (MD: -0.85; 95% CI: -1.76 to 0.07; P=0.07) (Fig 5).

**Comment 3:** In lines 260–263, the sentence "The meta-analysis showed that a significant improvement in knee function with NMES..." contains a structural error due to the unnecessary use of the word "that." Removing "that" improves grammatical accuracy and sentence flow.

**Reply 3**: Thank you for identifying this grammatical oversight. We have revised the sentence in lines 260–263 by removing the redundant "that," as recommended.

**Page 14, line 245-248 in red**: The meta-analysis showed a significant improvement in knee function with NMES (MD:4.46; 95% CI:2.08 to 6.84; p=0.0002; low certainty of evidence) with moderate statistical heterogeneity (I²=42%) compared to exercise only (Fig 6).

**Comment 4:** In line 281, the sentence "A total of two evaluated the effect of NMES on VMO/VL ratio (17, 26)" would be clearer if revised to include the word "studies." Adding this clarifies the subject of the sentence, improving precision and readability.

**Reply 4:** Thank you very much for your insightful feedback on improving the clarity of our manuscript. We sincerely appreciate your attention to detail and the constructive suggestions provided. We have revised the sentence in line 281 to include the word "studies" for clarity.

**Page 15, line 269 in red**: Two studies evaluated the effect of NMES on VMO/VL ratio.

We sincerely thank the reviewers for their thorough evaluation and valuable feedback during this second round of review. Your insightful comments and constructive suggestions have significantly enhanced the quality and rigor of our meta-analysis. We deeply appreciate the time and expertise you have contributed to improving our work, and we look forward to any further recommendations you may have to further refine this study.

**Reply to reviewer #4**

**Comment 1:** First, I would like to thank you for the opportunity to review this paper. The authors have made a great effort in putting this piece of work together. However, the manuscript is not ready for publication, and a major revision is necessary. Conducting a systematic review is not an easy task, especially when it is done in a second language. All my comments and suggestions are made solely with the purpose of improving the quality of this systematic review and encouraging the authors to enhance their skills in this type of study.

**Reply 1**: Thank you for your valuable review of our manuscript. We deeply appreciate the time and expertise you have dedicated to evaluating our work. Your constructive feedback is invaluable, and we are genuinely grateful for your thorough assessment.

Your comments have helped us recognize the significant shortcomings in our systematic review methodology and writing. Your insights have highlighted areas where our approach needed substantial improvement. We have carefully reviewed all your suggestions and have made comprehensive revisions throughout the manuscript accordingly. Your guidance has been instrumental in enhancing not only this particular paper but also our understanding of systematic review methodology more broadly.

**Comment 2**: One general comment I have concerns the readability of the English. Certain parts of the text are difficult to follow, and some terms are used inappropriately (e.g., ‘timing’ instead of ‘time points’). A useful approach to improve this would be to follow the structure of a high-quality systematic review when organising your own. Additionally, consistency in the use of terms and concepts is necessary (e.g., when evaluating the risk of bias). I also suggest changing the term ‘individuals’ to ‘people’.

**Reply 2:** Thank you sincerely for your careful review and feedback on our manuscript's overall writing and readability. After carefully consulting multiple high-quality systematic reviews, we have made diligent efforts to thoroughly revise the entire text, including the use of relevant terminology and concepts, to improve the clarity and quality of our paper.

**Comment 3**: Overall, the introduction is too lengthy. The authors should be more concise, focusing on previously published systematic reviews and clarifying what this review contributes to the existing PFP literature. What distinguishes this review from previous ones?

**Reply 3:** We sincerely thank the reviewer for this constructive feedback regarding our introduction. We have thoroughly revised it to make it more concise and coherent. Regarding the contribution of this review to the existing PFP literature and what distinguishes it from previous reviews, we have written a separate paragraph in the introduction to clearly state this.

**Comment 4**: Line 65-66: The authors state that PFP is a multifactorial condition, with risk factors categorised as extrinsic and intrinsic. However, this categorisation seems unusual, as it is difficult to determine which factors directly cause the onset of PFP symptoms. While it is known that people with PFP may present certain physical characteristics, such as muscle weakness, it is unclear whether these are causative factors for the onset of symptoms. Furthermore, it is not explained how equipment and environment contribute to the onset of PFP symptoms. What are the references supporting these claims? Additionally, how is tension considered an intrinsic risk factor for PFP? I suggest the authors restructure this part of the introduction and provide appropriate references for each statement.

**Reply 4:** We sincerely thank the reviewer for this insightful observation regarding our categorization of risk factors for PFP. After reviewing multiple relevant studies, we realized that categorizing PFP risk factors as intrinsic and extrinsic factors is not very accurate. We have now revised this section, referencing the statement from the International Patellofemoral Pain Research Retreat, to categorize them as distal, proximal, and local factors, with appropriate references provided for each factor. Regarding the term "tension," we apologize for not being clear. Here we are referring to the tension of the quadriceps and hamstring muscles, as research has shown that excessive tightness in these muscles is one of the risk factors for PFP.

**Page 3-4, line 60-69 in red**: Prospective research has identified that patellofemoral pain etiology can be categorized into proximal, local, and distal factors [2]. Proximal factors involve hip muscle weakness leading to altered lower limb kinematics during weight-bearing activities [3]. Local factors include quadriceps dysfunction [4], altered Vastus Medialis Oblique (VMO)/ Vastus Lateralis (VL) activation timing [5], quadriceps and hamstring tightness [6]. Distal factors encompass foot and ankle mechanics, such as increased navicular drop, which affect tibial rotation and subsequently alter patellofemoral joint mechanics [7]. All these factors contribute to PFP by creating abnormal patellofemoral joint mechanics and increased joint stress.

**Comment 5:** Line 69: The authors appear to place excessive emphasis on muscle delayed activation, which does not seem to be a particularly important factor in people with PFP. As PFP is a multifactorial condition, other factors, such as psychological influences, may also play a significant role.

**Reply 5:** We sincerely thank the reviewer for this insightful comment. We agree that our original text may have overemphasized this single factor without adequately acknowledging the complex, multifactorial nature of PFP. We have revised it to "altered VMO/ VL activation timing" and avoided suggesting it as the primary or most important factor.

**Page 3-4, line 63-65 in red**: Local factors include quadriceps dysfunction [4], altered Vastus Medialis Oblique (VMO)/ Vastus Lateralis (VL) activation timing [5], quadriceps and hamstring tightness [6].

**Comment 6:** Line 73: Please provide studies that support the claim that lifestyle changes are important for the treatment of PFP.

**Reply 6:** We appreciate the reviewer's careful assessment of our manuscript. After reviewing relevant literature, we believe that lifestyle changes can improve symptoms in PFP patients to some extent, but it is not one of the important and preferred treatment methods for PFP. Therefore, we have removed this expression from the manuscript.

**Page 4, line 70-73 in red**: The primary treatment for PFP is conservative, focusing on rehabilitation through exercise therapy, which is widely recognized as the main treatment for musculoskeletal conditions including PFP and is often combined with adjunct treatments to achieve optimal clinical outcomes [8, 9].

**Comment 7:** Line 75: I suggest changing ‘additional therapies’ to ‘adjunct treatments’.

**Reply 7:** Thank you for this helpful suggestion regarding terminology. We have made the corresponding changes in the manuscript.

**Page 4, line 72-73 in red:** often combined with adjunct treatments to achieve optimal clinical outcomes [8, 9].

**Comment 8:** Lines 76-78: Please provide reference to state that NMES may further enhance recovery.

**Reply 8:** Thank you for your valuable feedback regarding of our manuscript. We acknowledge the need to provide proper references supporting our statement that NMES may further enhance recovery. We have added the relevant references in the manuscript to support this viewpoint.

**Page 4, line 73-76 in red:** Neural muscular electrical stimulation (NMES), as a painless and non-invasive technique, is typically used as an adjunct treatment method to further promote recovery [10, 11].

10. Hauger AV, Reiman MP, Bjordal JM, Sheets C, Ledbetter L, Goode AP. Neuromuscular electrical stimulation is effective in strengthening the quadriceps muscle after anterior cruciate ligament surgery. Knee surgery, sports traumatology, arthroscopy : official journal of the ESSKA. 2018;26(2):399-410. Epub 2017/08/19. doi: 10.1007/s00167-017-4669-5. PubMed PMID: 28819679.

11.	Borzuola R, Laudani L, Labanca L, Macaluso A. Superimposing neuromuscular electrical stimulation onto voluntary contractions to improve muscle strength and mass: A systematic review. European journal of sport science. 2023;23(8):1547-59. Epub 2022/07/21. doi: 10.1080/17461391.2022.2104656. PubMed PMID: 35856620.

**Comment 9:** Lines 84-95: The authors cite three clinical trials in the introduction to support their review. However, it would be more appropriate to reference systematic reviews that have already synthesised this evidence. Existing reviews generally indicate that the effects of using NMES are inconclusive. Citing such reviews would strengthen the introduction and save words.

**Reply 9:** Thank you for this valuable feedback. According to the feedback, we have revised this section, removed citations of individual clinical trials and instead citing high-quality systematic reviews to indicate that whether NMES combined with exercise therapy can produce better clinical benefits for PFP patients remains uncertain.

**Page 4-5, line 77-87 in red:** However, whether NMES combined with exercise therapy can produce better clinical benefits for PFP patients currently has no definitive conclusion. A recent systematic review indicated that NMES combined with exercise therapy can slightly improve pain in patients with PFP, but does not significantly improve knee function. However, the evidence certainty of these results is very low[14]. Furthermore, the review did not evaluate key indicators such as muscle strength and lacked subgroup analysis based on intervention duration. Given the emergence of more evidence and the weakness of existing evidence, further systematic reviews are necessary to clarify the clinical value of NMES as an adjunct treatment for patients with PFP.

**Comment 10:** Recently, Souto et al. published a systematic review evaluating the effectiveness of various adjunct treatments combined with exercise therapy on self-reported pain and functional outcomes. How do the results of the present review differ from those reported by Souto et al.? Has new evidence emerged since that review was published? This should be clarified in the introduction.

**Reply 10:** We appreciate the reviewer's thoughtful comment. Our system evaluation results differ from those reported by Souto et al., with specific details discussed in the paper's discussion section. Additionally, compared to Souto et al.'s system evaluation, we included more literature and added two new outcome measures: quadriceps strength and VMO/VL activation ratio. We also conducted subgroup analysis based on intervention time to determine the impact of different intervention durations on PFP patients.

**Comment 11:** Why did the authors not cite the Cochrane Handbook for Systematic Reviews of Interventions? It is a fundamental reference for conducting and reporting systematic reviews.

**Reply 11:** Thank you for your valuable feedback regarding our citation practices. We acknowledge this oversight in our manuscript. The Cochrane Handbook is recognized as a gold standard resource that provides comprehensive methodological guidance for conducting and reporting systematic reviews. In the revised manuscript, we have added citations to the Cochrane Handbook in the Methods section.

**Page 5 line 95-97 in red:** This systematic review follows the Methodological Expectations of Cochrane Intervention Review standards [15], Preferred Reporting Items for Systematic Reviews and Meta-Analyses (PRISMA) [16].

**Comment 12:** Lines 118-121: Type of study: The English in this section needs improvement, as it is difficult to follow. Please revise for clarity and coherence.

**Reply 12:** We sincerely appreciate your careful reading and constructive feedback regarding the clarity of the "Type of study" section. We have revised this section to enhance clarity and ensure better readability. The revised text now provides a more coherent description of this section.

**Page 6 line 111-112 in red:** This review only included randomized controlled trials published in peer-reviewed journals, with no restrictions regarding language or publication date.

**Comment 13:** Lines 123-126: Type of participants: What criteria were used to diagnose PFP? Was it consistent with the 2016 PFP consensus guidelines?

**Reply 13:** We appreciate the reviewer's important question regarding the diagnostic criteria for patellofemoral pain (PFP) in our systematic review. We acknowledge that we did not explicitly state whether all included studies followed the 2016 PFP consensus guidelines. Upon review, some of the included studies were published before these guidelines were established, but generally followed similar diagnostic approaches. For studies published after 2016, we have now verified their alignment with these consensus guidelines were reported. We have revised this section to clarify the diagnostic criteria used in the included studies.

**Page 6 line 114-115 in red:** We included studies of adults with a diagnosis of PFP according to international consensus statements [1].

**Comment 14:** Lines 128-134: Type of interventions: Please review the English in this section for clarity and coherence. Were studies evaluating an NMES sham included?

**Reply 14:** We sincerely appreciate your valuable suggestions regarding improving the clarity and coherence of the intervention types section. We have thoroughly revised this section to enhance its clarity and coherence. Furthermore, in the revised manuscript, we have explicitly stated that studies utilizing exercise therapy combined with NMES sham treatment as the control intervention were also included for evaluation

**Page 6 line 119-121 in red:** In the study, the experimental group received exercise therapy combined with NMES, while the control group only received exercise therapy or exercise therapy combined with sham NMES treatment.

**Comment 15:** Line 144: Please specify what is meant by “isometric strength assessments.”

**Reply 15:** Thank you for your thorough review and valuable comments on our manuscript. Regarding your suggestion about line 144 concerning the need to specify what is meant by "isometric strength assessments," we appreciate your attention to detail. I would like to clarify that this was an inaccurate expression in our original manuscript. What we intended to convey was that quadriceps strength was determined through both isometric and isokinetic strength assessment methods. In the revised version, we have corrected this statement.

**Page 7 line 127-129 in red:** The strength of the quadriceps muscle was determined through isometric or isokinetic strength assessment.

**Comment 16:** Line 157: Data extraction: Why is the study design being extracted if only RCTs were included?

**Reply 16:** Thank you for your insightful comment. You correctly pointed out that this creates redundancy since we had already specified that only RCTs were included in our meta-analysis on the effects of NMES on patients with PFP. We agree with your observation and have addressed this issue by removing the mention of "study design" from the data extraction section in our revised manuscript. This elimination prevents unnecessary repetition and improves the conciseness of our methodology description.

**Page 7 line 141-145 in red:** (1) Name of the leading author; the publication year; and the country of the research. (2) The sample size; the age range of participants; and the gender distribution. (3) Details about the intervention program, including its duration; the frequency, duration, and intensity of NMES sessions. (4) The outcome data collected and the timing of measurements.

**Comment 17:** The authors conducted a risk of bias assessment, not a methodological quality or quality assessment. Consistency in terminology is necessary throughout the manuscript.

**Reply 17:** Thank you for your valuable feedback regarding the inconsistent terminology in our manuscript. We agree that consistency in terminology is essential for clarity and scientific rigor. As you correctly pointed out, we conducted a risk of bias assessment rather than a methodological quality or quality assessment. We have made corresponding modifications to the title of this section and its content to ensure consistency of terminology throughout our paper.

**Page 8 line 147-150 in red:**

2.4. Risk of bias assessment

The revised Cochrane Risk of Bias tool for randomized trials was used by two independent researchers (ZJW and WZX) to assess the risk of bias of the included studies [17].

**Comment 18:** Provide the full name of GRADE (Grading of Recommendations, Assessment, Development and Evaluations) before using the abbreviation.

**Reply 18:** Thank you for your valuable suggestion. We have revised our manuscript to provide the full name "Grading of Recommendations, Assessment, Development and Evaluations (GRADE)" before using the abbreviation throughout the text. This change ensures clarity for all readers and follows proper scientific writing conventions.

**Page 8 line 158-163 in red:** The assessors (ZJW, WZX) appraised the evidence's reliability for the outcomes by employing the Grading of Recommendations, Assessment, Development and Evaluations(GRADE) approach. This approach takes into account five criteria (risk of bias, inconsistency, indirectness, imprecision, and publication bias) to ascertain the reliability of the evidence, categorizing it into four levels (high, moderate, low, very low) [18].

**Comment 19:** Lines 186-189: Statistical analysis: Clearly state that I² assesses statistical heterogeneity. A random-effects model should be applied based on methodological heterogeneity, not statistical heterogeneity. How did the authors classify statistical heterogeneity, and what cut-offs were used? I recommend that the authors consult again the Cochrane Handbook for guidance.

**Reply 19:** Thank you for your valuable comments regarding our statistical analysis methodology. Regarding the classification of statistical heterogeneity, we followed the guidance from the Cochrane Handbook for Systematic Reviews of Interventions. Specifically, we defined statistical heterogeneity as: 30-50% representing moderate heterogeneity, 50-75% representing substantial heterogeneity, and >75% representing considerable heterogeneity. We have added this clarification to the statistical analysis section. Concerning the application of a random-effects model, we agree with your point that this decision should primarily be based on methodological heterogeneity rather than statistical heterogeneity alone. Considering the differences in intervention methods among the included studies and the limited number of studies, we uniformly applied a random-effects model for analysis and made corresponding in the results section.

**Page 9 line 172-176 in red:** Moderate, substantial, and considerable statistical heterogeneity were defined by I² values of 30%, 50%, and 75%, respectively [15]. Random effects model was used for analysis due to differences in intervention methods between studies and the limited number of studies [20].

**Comment 20:**Line 197: How was the ‘intervention time’ classified—short, medium, or long? Please clarify by specifying the duration associated with each category.

**Reply 20:** Thank you for your valuable comment. According to relevant literature and the intervention durations of included studies, we defined ≤4 weeks as short-term and 4–12 weeks as medium-term. Additionally, we have modified the original classifications of "<1 month" and "1–3 months" in other sections of the article.

**Page 9 line 181-182 in red:** with ≤4 weeks defined as short-term and 4-12 weeks defined as medium-term [21]**.**

**Comment 21:** Line 206: English-language database? However, papers not published in English were also included in these databases. I recommend excluding it.

**Reply 21:** We appreciate the reviewer's careful assessment of our manuscript. Regarding this point, we need to clarify that although Wu et al.'s research was published in Chinese, we obtained it through the Embase database search, and this study meets our inclusion criteria. Furthermore, as stated in "2.1.1. Type of study": there were no restrictions regarding language or publication date. Therefore, we did not exclude this paper.

**Comment 22:** Line 209: Did the authors attempt to contact the researchers of the unavailable studies? If so, how many attempts were made? Was the standard of three or more attempts followed?

**Reply 22:** We appreciate the reviewer's careful assessment of our manuscript. For studies where full texts were unavailable, we did attempt to contact the original authors, but we did not strictly adhere to the standard of three contact attempts. In our methodology section, we will add this information to clearly explain our efforts to obtain full texts. Studies that remained inaccessible were excluded from our final analysis, and these exclusions were indicated with appropriate reasons in our study flow diagram.

**Page 10 line 192-194 in red:** Two studies were excluded due to the inability to obtain valid data and the lack of effective responses after contacting the authors.

**Comment 23:** Lines 209-210: Were nine studies included in this meta-analysis or the overall review? Were all nine studies included in the meta-analysis? Please clarify.

**Reply 23:** We appreciate your attention to this detail. In the revised manuscript, we have clarified that all nine studies identified in our systematic review were included in our meta-analysis. There was no difference between the studies included in the overall review and those analyzed quantitatively.

**Page 10 line 194-195 in red:** A total of 9 studies were ultimately included, all of which underwent quantitative analysis.

**Comment 24:** Line 216: Please write ‘United States’ instead of ‘US’ to avoid unnecessary abbreviations. Table 1: Remove the study design, as it is already clear that only RCTs were included. Use IC and CG instead of I and C for improved clarity. Additionally, clarify what QS stands for.

**Reply 24:** Thank you for your helpful suggestions regarding terminology and table contents in our manuscript. Following your suggestions, we have made the relevant modifications and added a footnote to the table clarifying that "QS" stands for "Quadriceps muscles strengthened".

**Comment 25:** Lines 234-240: Risk of bias and study quality: Ensure consistency in terminology throughout. This paragraph is very unclear — risk of bias should be assessed as ‘low risk’, ‘some concerns’, or ‘high risk’. I recommend mentioning the certainty of the evidence only alongside the pooled results, as you have done elsewhere.

**Reply 25:** Thank you for your valuable feedback regarding the risk of bias assessment and terminology consistency in our manuscript. We have revised the paragraph on risk of bias and study quality. As you correctly pointed out, we have now standardized our risk of bias assessments using "low risk," "some concerns," and "high risk" to describe the bias risk results of the included studies. We have also restructured the presentation of evidence certainty, removing it from this section and only presenting it alongside the pooled results, as you suggested. These changes have improved the clarity and consistency of our risk of bias reporting.

**Page 13 line 220-224 in red:**

4.3. Risk of bias

Figures 2 and 3 show the risk of bias for each study. We assessed four studies as low risk [25, 27, 29, 31], two studies as having some concerns [23, 26], and the other three studies as high risk [24, 28, 30]. The bias was primarily due to the implementation of blinding methods and incomplete outcome data.

**Comment 26:** Line 249: The authors mention the level of statistical heterogeneity without having specified it previously in the statistical analysis section. Please provide a clear definition and explanation of how statistical heterogeneity was assessed in the statistical analysis section.

**Reply 26:** Thank you for your careful reading and for highlighting this important oversight in our manuscript. We have revised the statistical analysis section to include a clear definition and explanation of how statistical heterogeneity was assessed, and in the results section, we have modified the description of heterogeneity magnitude according to the definition.

**Comment 27:** Lines 259-260: Was the abbreviation AKPS introduced earlier in the manuscript? If so, it can be used here; otherwise, provide the full term before using the abbreviation. In the results section, always clearly specify which intervention was being compared to what. For example: The meta-analysis showed that a significant improvement in knee function with NMES (MD:4.75; 95% CI:3.04 to 6.46; p<0.00001; low certainty of evidence) with moderate heterogeneity (I²=42%) compared to exercise only. (Fig 6).

**Reply 27:** Thank you for your valuable feedback. Regarding the issue with the AKPS abbreviation, since we have already mentioned it in the previous section 2.1.1. Types of outcome measures, we therefore used the abbreviation here. For the results section, we have rewritten similar statements as per your suggestion to clearly specify which intervention was being compared to what intervention. These changes will improve the clarity and readability of our manuscript. We appreciate your careful review and constructive suggestions.

**Comment 28:** Lines 288-290: Publication bias: You cannot conclude that there is no evidence of bias when each outcome involves fewer than nine trials. In fact, it is more accurate to state that publication bias could not be assessed due to the limited number of trials per outcome.

**Reply 28:** Thank you for your feedback regarding our assessment of publication bias. We have revised our statement about publication bias to accurately reflect the limitations of our analysis. Instead of concluding there is no evidence of bias, we will clearly state: "Publication bias could not be properly assessed due to the limited number of trials per outcome (fewer than nine trials for each outcome)." We appreciate your careful review, which has helped improve the accuracy of our paper.

**Page 15 line 277-278 in red:** Publication bias could not be assessed due to the limited number of trials per outcome.

**Comment 29:** You cannot claim that NMES can effectively enhance pain, function, and strength, as your findings are based on very low to low certainty of evidence.

**Reply 29:** Thank you for your important feedback regarding the strength of our conclusions in relation to the certainty of evidence. We will revise our conclusion to more accurately reflect the limitations of our findings. Instead of claiming that NMES "can effectively enhance pain, function, and strength," we will modify our wording to acknowledge the uncertainty, stating that "NMES may potentially improve pain, function, and strength outcomes, though this finding is based on very low to low certainty evidence, suggesting the need for further high-quality research before drawing definitive conclusions."

**Page 16 line 286-289 in red:** However, it should be emphasized that these findings are still based on low-quality evidence, and although the level of evidence has slightly improved compared to previous studies, it remains insufficient to draw definitive conclusions.

**Comment 30:** “Previous studies have suggested that NMES may reduce pain in PFP, but the evidence in earlier systematic reviews was limited and characterized by low certainty.” Your findings do not differ from those reported in previous studies. Additionally, you have not clearly reported the risk of bias for each study. How many studies were assessed as having ‘low risk’?

**Reply 30:** Thank you for your important feedback regarding our conclusions and risk of bias reporting. Regarding our findings related to prior research: While we found pain relief effects similar to previous studies, our expanded sample and additional subgroup analysis based on intervention duration provide incremental evidence rather than identical conclusions. Furthermore, for knee function, we reached different results from previous systematic reviews after expanding our sample size. We also additionally evaluated the effects of NMES combined with exercise therapy compared to exercise therapy alone on quadriceps strength and VMO/VL activation ratio in PFP patients, as well as the impact of different intervention durations, which previous studies have not reported. Of course, considering the evidence level is low or very low, we will adjust our language to avoid exaggerating the novelty of our findings. Regarding the risk of bias reporting: We appreciate you highlighting this lack of clarity. We have modified and supplemented this section in our revised manuscript. We thank you for helping us improve the accuracy and clarity of our reporting. These revisions will help ensure the rigor of our conclusions.

**Comment 31:** You should revise your entire discussion, considering that your findings are based on very low to low certainty of evidence. Additionally, avoid using the term ‘treatment’; instead, use ‘exercise therapy’, as that is the focus of your systematic review.

**Reply 31:** We greatly appreciate the reviewer's valuable suggestions. In light of the certainty level of our evidence, we have thoroughly revised the discussion section and reported this in the limitations section, avoiding overstatement of the effectiveness of NMES combined with exercise therapy for PFP patients, ensuring that our conclusions are consistent with the quality of evidence. Additionally, we have replaced all instances of the term "treatment" with "exercise therapy" throughout the manuscript to more accurately reflect the focus of our systematic review. These modifications make our discussion more rigorous and precise, avoiding potential overinterpretation. We again thank the reviewer for their guidance, which is crucial for improving the quality of our research.

We would like to express our profound gratitude to the reviewer for the exceptionally detailed and comprehensive feedback provided during the review. Your meticulous examination of our meta-analysis and the numerous thoughtful suggestions have been invaluable in refining our methodology, strengthening our statistical analysis, and improving the overall clarity of our manuscript. We have diligently addressed each point raised and believe these revisions have transformed our paper substantially. We sincerely appreciate your significant contribution to enhancing the quality of our research, and we look forward to any further recommendations you may have to further refine this study.

**Reply to reviewer #5**

**Comment 1:** The purpose of this study was to investigate the effects of adding neuromuscular electrical stimulation (NMES) to exercise therapy on pain, knee function, quadriceps strength, and the ratio of activation of the Vastus Medialis Obliqus (VMO) to Vastus Lateralis (VL) muscles in people with Patellofemoral pain (PFP). The manuscript’s theme is adequate for publication in PLOS ONE, it is relevant and has novelty for the area of NMES on the rehabilitation of patients with patellofemoral pain. The main findings of the study were that NMES superimposed to exercise therapy reduced pain, improved knee function and increased quadriceps muscle strength when the treatment period was longer than one month compared to exercise therapy alone. The introduction leads the reader to the study problem. The literature reviewed is adequate and updated. However, minor adjustments are needed (Please see Specific Comments). The methods section is relatively clear, but minor changes are needed. The statistical analysis is ok. The results are adequately described and presented in one table and 10 figures. The discussion is clear, and objective and the data support the study conclusion. The references section is adequate. The manuscript was presented in an intelligible fashion and written in Standard English. Minor changes are needed in the manuscript that are described below, and which might help to improve the manuscript’s clarity and quality.

**Reply 1:** We acknowledge your positive comments regarding our introduction, literature review, statistical analysis, results presentation, and discussion. We have revised the manuscript according to your feedback, and we believe these changes have further enhanced the quality and standard of our article.

**Comment 2:** Lines 37-38: The number of participants is not correct: 171+166=337 and not 360. Please correct.

**Reply 2:** Thank you for pointing out this mathematical error. We are grateful for your careful review that identified the incorrect calculation in lines 37-38 where we stated that 171+166=360 instead of the correct sum of 337. We apologize for this oversight and have corrected this mistake in the revised manuscript. We appreciate your thorough attention to detail which helps improve the accuracy of our paper.

**Page 2 line 37-38 in red:** Nine randomized controlled trials, encompassing 337 participants (171 intervention, 166 control), were included in the analysis.

**Comment 3:** Line 67. To which equipment are you referring to? Physical fitness equipment, equipment used at work?

**Reply 3:** Thank you for your careful review of our manuscript. Regarding this section on risk factors for PFP in the background, we have made revisions based on feedback from other reviewers. We have referenced the statement from the International Patellofemoral Pain Research Retreat and changed it to "distal, proximal, and local factors." As for the equipment, we have removed it.

**Page 3-4, line 60-69 in red**: Prospective research has identified that patellofemoral pain etiology can be categorized into proximal, local, and distal factors [2]. Proximal factors involve hip muscle weakness leading to altered lower limb kinematics during weight-bearing activities [3]. Local factors include quadriceps dysfunction [4], altered Vastus Medialis Oblique(VMO)/ Vastus Lateralis(VL) activation timing [5], quadriceps and hamstring tightness [6]. Distal factors encompass foot and ankle mechanics, such as increased navicular drop, which affect tibial rotation and subsequently alter patellofemoral joint mechanics [7]. All these factors contribute to PFP by creating abnormal patellofemoral joint mechanics and increased joint stress.

**Comment 4:** Line 68. While strength and force are usually related to muscle function, tension is usually used in bone, ligament, tendon and cartilage function, as they passively generate tension to support the forces produced by muscle contraction or external forces applied to the human body. Perhaps specifying which tension you are referring to here will help the reader to better understand the intrinsic risk factors for the development of PFP.

**Reply 4:** We appreciate the reviewer’s insightful comment regarding the use of the term *tension*. We apologize for any lack of clarity in our original manuscript. In this context, we are specifically referring to muscle tension in the quadriceps and hamstrings, as research suggests that excessive tightness in these muscles can alter patellofemoral joint mechanics and contribute to the development of PFP .We have revised the text to clarify this point . Thank you for highlighting this important distinction between muscle-generated tension and the passive tension found in bones, ligaments, tendons, and cartilage.

**Page 3, line 65 in red**: quadriceps and hamstring tightness [6].

**Comment 5:** Lines 78-79. NMES stimulates not motor nerves, but motor and sensory neurons, as most nerves are composed of both sensory and motor neurons. Discomfort is mostly a result from the activation of sensory receptors at the skin, tendon and ligament receptors and activation of sensory neurons by NMES. But here you need to change the word “nerve” by “neurons”.

**Reply 5:** Thank you for this important clarification. We will revise our manuscript to replace "nerve" with "neurons" to more accurately reflect the physiological mechanisms of NMES. This precision in terminology will help ensure our paper correctly describes the neurophysiological mechanisms involved in NMES. This change will be implemented throughout the manuscript to maintain consistency in our terminology.

**Page 4, line 76-77 in red:** NMES works by stimulating motor neurons, leading to muscle contractions that strengthen muscles [12, 13].

**Comment 6:** Lines 120-121. The sentence is missing something here, such as “were applied”.

**Reply 6**: We sincerely appreciate your careful reading and comments on our writing. Regarding the "Type of study" section, we acknowledge that the English in this section needs improvement. We have revised this section to enhance clarity and ensure better readability. The revised text now provides a more coherent description of this section.

**Page 6, line 111-112in red:** This review only included randomized controlled trials published in peer-reviewed journals, with no restrictions regarding language or publication date.

**Comment 7:** Line 139: Please correct the name of the muscle: Vastus Medialis Oblique.

**Reply 7:** Thank you for your careful attention to anatomical terminology in our manuscript. In our revised manuscript, since Vastus Medialis Oblique has already appeared earlier in the text, we have used the abbreviation VMO here. We have also ensured the correct spelling when this term first appears. We appreciate your expertise in identifying this error, as precise anatomical terminology is essential for the scientific quality of our paper.

**Comment 8:** Line 144: Please specify what you mean by other isometric strength assessments

**Reply 8:** Thank you for your thorough review and valuable comments on our manuscript. I would like to clarify that this was an inaccurate expression in our original manuscript. What we intended to convey was that quadriceps strength was determined through both isometric and isokinetic strength assessment methods. In the revised version, we have corrected this statement.

**Page 7 , line 127-129 in red:** The strength of the quadriceps muscle was determined through isometric or isokinetic strength assessment.

**Comment 9:** Line 156. Name of the leading author.

**Reply 9:** Thank you for bringing this to our attention. In the revised manuscript, we have changed "the name of the lead author" to "Name of the leading author." We appreciate your attention to detail to ensure the professionalism of our paper.

**Page 7 , line 141 in red:** (1) Name of the leading author;

**Comment 10:** Line 206. In the abstract you refer to five databases and here you refer to four databases. Which is the correct one?

**Reply 10:** Thank you for your careful review, and we sincerely apologize for our oversight. We searched a total of five databases, as illustrated in the PRISMA flowchart. We have revised the manuscript accordingly to ensure consistency and rigor throughout the paper.

**Page 10 , line 190 in red:** We searched five English-language databases, retrieving 290 studies.

**Comment 11:** Lines 214-215. Like the comment made for the abstract, the number of participants is not correct: 171+166=337 and not 360. Please correct.

**Reply 11:** Thank you for bringing this discrepancy to my attention. We have made the corresponding revisions in the revised manuscript to ensure consistency throughout the paper and the accuracy of the reported sample size.

**Page 10, line 200-201 in red:** Nine randomized controlled trials with 337 participants were included (171 in the experimental group, 166 in the control group) [23-31].

**Comment 12:** Lines 217-218. I think that you are missing two studies here regarding the duration of the interventions.Table 1. I would reorganize the studies in the table alphabetically by the authors’ last names, as this makes it easier for the reader to find the studies. In addition, you need to use only the last name of the first author with the name with the first letter capitalized (e.g., Akarcali et al., 2013; Bily et al., 2008; Celik et al., 2019; Das et al., 2016; Glaviano et al., 2020; Jing et al., 2024; Kumar et al., 2023; Mv et al., 2023; Nie et al., 2024; Talbot et al., 2020).Please, also correct the abbreviation NMES in the table. I also suggest that all the abbreviations are presented alphabetically, as this helps the reader to find the description of each abbreviation easily.

**Reply 12:** Thank you for your thorough review and constructive feedback. We appreciate your attention to detail. Regarding the description of intervention durations across studies, after carefully reviewing the original manuscript, we did not find any studies that were omitted. As for Table 1, we will implement all your suggestions. In the revised manuscript, we have rearranged the included studies and abbreviations in the notes in alphabetical order by the last name of the first author for easier reference, and we have also corrected the NMES abbreviation in the table. These changes will enhance the clarity and readability of our manuscript. Thank you for helping us improve the quality of our paper.

**Page 12, line 218 in red:** Table 1. Characteristics of the included studies

**Comment 13:** Line 270. You mention six studies, but you show only five within the parentheses.

**Reply 13:** Thank you for pointing out this discrepancy. I apologize for the carelessness in our manuscript. After careful verification, we confirm that there are indeed only 5 studies cited in the parentheses, not 6 as incorrectly stated in the text. We have corrected this inconsistency in the revised manuscript to ensure accuracy. We appreciate your thorough review which helped us identify and address this error.

**Page 14, line 256 in red:** Five studies assessed quadriceps strength[23-26, 28].

**Comment 14:** Line 281. “Two studies evaluated the effect…”

**Reply 14:** Thank you for your valuable comment. We have revised the sentence to “Two studies evaluated the effect of NMES on VMO/VL ratio [25, 31]” as suggested, to improve clarity and conciseness.

**Page 15, line 269 in red:** Two studies evaluated the effect of NMES on VMO/VL ratio [25, 31].

**Comment 15:** Lines 302-304. It is not clear in your explanation regarding the pain gating theory why by stimulating A-α and A-β nerve fibers, NMES reduces the pain transmitted by pain receptors. You also need to specify which nerve fibers do transmit pain signals so that the reader understands the difference between nerve fibers that transmit and those that do not transmit pain stimuli.

**Reply 15:** Thank you for your insightful comment. We have revised the explanation of the pain gating theory to clarify that NMES reduces pain by stimulating large-diameter A-α and A-β fibers, which activate inhibitory interneurons in the dorsal horn of the spinal cord. This process suppresses the transmission of pain signals carried by smaller-diameter A-δ and C fibers. The revised text now clearly distinguishes between the nerve fibers responsible for pain inhibition and those that transmit pain stimuli. This clarification helps improve the scientific accuracy and comprehensibility of the manuscript, making the underlying mechanisms of NMES-induced analgesia more accessible to readers.

**Page 16, line 296-299 in red:** NMES preferentially stimulates large diameter, myelinated A-α and A-β fibers that transmit non-noxious signals and produce competitive inhibition of pain signals transmitted by smaller A-δ and C fibers. This effectively "closes the gate" on pain transmission, reducing the perception of knee pain.

**Comment 16:** Lines 316-318. This sentence is confusing as you start referring to the fact that exercise therapy alone may reach a plateau or ceiling effect, and then you continue saying that the nervous system adapts to the NMES stimulus. Is the ceiling effect related only to exercise therapy or to both exercise therapy and NMES? As you started this paragraph calling the reader’s attention to the fact that your study found that NMES contributes to improved knee function, it appears to me that you are discrediting your results while contrasting them to the existent literature. Which one should the reader follow: NMES really contributes to improving knee function or does NMES not really contribute to knee function?

**Reply 16:** Thank you for your thoughtful and constructive comment. Regarding this section, our intention was to emphasize that although our findings, based on a more comprehensive inclusion of studies, differ from previous systematic reviews and individual studies in terms of the effect of NMES combined with exercise therapy versus exercise alone on knee function in patients with PFP, the result may not be entirely reliable. However, we acknowledge that our original expression may have caused confusion, making it difficult for readers to understand. Therefore, we have thoroughly revised this part in the revised manuscript. First, we clarified that NMES combined with exercise therapy may be more effective than exercise alone in improving knee function in patients with PFP, and we explained the possible underlying mechanisms. We then noted that due to variations in NMES parameters among the included studies and the low certainty of evidence, further high-quality research is needed to confirm this conclusion.

**Page 17, line 303-315 in red:** Our research found that NMES combined with exercise therapy can further improve knee joint function in PFP patients, which may be related to NMES's ability to relieve pain and increase VMO activation levels[33, 34]. Pain reduction enables patients to participate more actively in treatment, enhancing the therapeutic effect of exercise therapy, while increased VMO activation helps improve patellar mechanical trajectory and stability[35]. These synergistic effects collectively enhance the recovery of knee joint function in PFP patients. However, our results are not consistent with previous systematic reviews and some studies[14, 23, 26]. Possible reasons for this discrepancy may be variations in NMES parameters across studies, such as frequency, duration, and equipment, which could affect treatment outcomes. Given the low-certainty evidence of this result, and the heterogeneity of NMES parameters between included studies, more high-quality RCTs are needed in the future to verify the effect of NMES combined with exercise therapy on knee joint function in PFP patients.

**Comment 17:** Line 324. I think that you are using here PT as an abbreviation for Physical Therapy. However, in previous paragraphs you used physical therapy, not the abbreviation. It is also not clear to me why you use in most of the manuscript the expression exercise therapy and not physical therapy. I would perhaps use the term physical therapy in the beginning (with the abbreviation PT after the first mention) and make it clear that you are referring to studies that used exercises during physical therapy. On the other hand, if you want to maintain the term exercise therapy, then perhaps do not use physical therapy in any part of the manuscript.

**Reply 17:** Thank you for your careful review and helpful suggestion. To maintain consistency and avoid confusion, we have revised the manuscript by replacing all instances of "physical therapy (PT)" with "exercise therapy." This ensures terminological consistency throughout the text and aligns with our focus on studies specifically involving exercise-based interventions. We appreciate your attention to clarity and terminology, which has helped improve the overall coherence of the manuscript.

**Comment 18:** Lines 327-331. You refer here to prior research, but it is not clear if you are referring only to reference 38. In addition, you may need to better describe how NMES superimposed to voluntary exercises may increase muscle force production. While during voluntary exercises the central nervous system recruits motor units through Henneman’s Size Principle from the smaller to the larger motor units, NMES recruits motor units that are closer to the electrodes placed on the skin, but with a preferential recruitment of larger motor units (constituted of type II fast-twitch muscle fibers) due to the lower resistance of the large diameter of their axons compared to the higher resistance of the smaller (type I slow-twitch muscle fibers) motor units’ axons. In other words, during exercise therapy larger motor units are probably not recruited due to the submaximal level of the exercises, and superimposing NMES will lead to the recruitment of large motor units that are not recruited during the voluntary exercises.

**Reply 18:** Thank you for your detailed and insightful comments. Your professional advice has not only deepened our understanding of the fundamental mechanisms of NMES, but also significantly improved the clarity and quality of our manuscript. Firstly, we have cited additional references, and secondly, we have provided a more detailed explanation of how NMES, when combined with exercise therapy, may enhance muscle strength generation. We appreciate your contribution to improving the scientific content and overall quality of the manuscript.

**Page 17-18, line 322-329 in red:** While voluntary contractions follow Henneman's Size Principle, where the central nervous system recruits motor units in order from small to large, NMES preferentially activates larger motor units dominated by type II fast-twitch fibers near the electrodes due to their lower resistance [41]. During submaximal exercise therapy, these larger motor units typically remain inactive. When NMES is superimposed on voluntary exercise, it recruits these otherwise inactive larger motor units, potentially explaining the enhanced force production observed with combined therapy [42].

**Comment 19:** Another aspect that you may add regarding the exercise therapy time and the NMES effects is that usually muscle adaptations occur after four weeks but with higher increases occurring after eight weeks of treatment. Therefore, 8-12 weeks will show a higher clinical effect compared to short-term (i.e., up to 4 weeks) exercise therapy.

**Reply 19:** Thank you for your valuable comment. In response to your suggestion, we have added this content to the discussion section regarding the subgroup analysis results in the manuscript. This addition helps to better explain the findings of the subgroup analysis, specifically that, compared to short-term treatment, when the intervention duration exceeds 4 weeks, NMES combined with exercise therapy can lead to better clinical outcomes for PFP patients. Once again, we sincerely appreciate your valuable advice.

**Page 18, line 340-344 in red:** Additionally, muscle adaptations typically begin to appear after 4 weeks, but only reach significant enhancement after 8 weeks, therefore, compared to short-term treatment, NMES can demonstrate more pronounced clinical effects only when treatment continues for more than one month[50, 51].

**Comment 20:** Finally, you may want to read manuscripts about wide-pulse NMES (i.e., with a larger pulse duration of 1 ms per pulse phase). This NMES type also recruits motor units via reflex pathways, thereby increasing the number of motor units recruited, which will generate a higher force production. In all the studies you showed in the review, pulse duration was short (65-400 μs) and is a limitation, and if you want to improve the NMES effects on muscle strength superimposed on voluntary exercises, new electrical stimulators with larger pulse duration also need to be introduced in clinical practice.

**Reply 20:** We appreciate your thoughtful comment, which has made us more aware of the influence of NMES pulse duration on muscle strength enhancement. After reviewing the relevant literature, we have gained a more comprehensive understanding of this aspect. As you rightly pointed out, the studies included in our review all used relatively short pulse durations. We have mentioned this limitation in the revised manuscript, noting that it may potentially obscure the clinical benefits of NMES combined with exercise therapy. Furthermore, we suggest that future research should further investigate NMES parameter settings to provide more robust evidence supporting the value of NMES combined with exercise therapy for patients with PFP.

**Page 19, line 353-358 in red:** and the current relevant research has relatively short pulse widths, while studies show that wider pulses are more conducive to motor unit recruitment, which may mask the intervention effect of NMES combined with exercise therapy[52]. Future studies should address these issues, include more high-quality trials, and establish standardized protocols for NMES combined with exercise therapy, to provide more reliable evidence supporting the value of NMES combined with exercise therapy in PFP patients.

**Comment 21:**

REFERENCES
Please review the references as I think that some are missing some data. I am also not sure about the format of the journals’ names, but I usually use all journal names with the first letter of each word capitalized.

**Reply 21:** Thank you for your careful review and constructive feedback on the references. In accordance with the journal’s reference formatting requirements, we have thoroughly checked all references in the revised manuscript and added any missing information to ensure their completeness and accuracy. We appreciate your attention to detail, which has helped us improve the overall quality and consistency of the manuscript.

**Comment 22:**

GENERAL COMMENT
I would add a space between the parentheses of the references and the words preceding the parentheses in all the text.

**Reply 22:** Thank you for your helpful comment. We have carefully reviewed the entire manuscript and ensured that a space has been added between the parentheses of the references and the preceding words, in accordance with formatting guidelines. We appreciate your attention to detail, which has contributed to improving the overall readability and presentation of our manuscript.

We extend our heartfelt gratitude to the reviewer for the exceptional feedback that has substantially elevated the quality of our manuscript. Under your expert guidance, our meta-analysis has been significantly strengthened, particularly in the discussion section. The comprehensive improvements made possible by your detailed suggestions have enhanced not only the scientific rigor but also the clinical relevance of our work. We deeply value your contribution to this research and eagerly anticipate any further recommendations you may provide to further refine this manuscript.

Yours sincerely,

Jian Chen

E-mail: 2007021@whsu.edu.cn
